# Optimizing Kidney Replacement Therapy During the COVID-19 Pandemic Across a Complex Healthcare System

**DOI:** 10.3389/fmed.2020.604182

**Published:** 2020-12-22

**Authors:** Jane Akomeah, Aljenica Apostol, Esteen Barnes, Chaim Charytan, Uvannie Enriquez, Madhavi Katikaneni, Frank Liu, Albert Messina, Kotresha Neelakantappa, Jai Radhakrishnan, Ritesh Raichoudhury, Ramya Ramakrishnan, Sadia Saboor, Alina Sapozhnikova, Jeffrey Silberzweig, Jacob S. Stevens, Susan Tanzi-Pfeifer, Jennifer Tutone, Vesh Srivatana

**Affiliations:** ^1^NewYork-Presbyterian/Columbia University Medical Center, New York, NY, United States; ^2^NewYork-Presbyterian Lower Manhattan Hospital, New York, NY, United States; ^3^NewYork-Presbyterian Queens, New York, NY, United States; ^4^NewYork-Presbyterian/Weill Cornell Medical Center, New York, NY, United States; ^5^NewYork-Presbyterian Hudson Valley Hospital, Cold Spring, NY, United States; ^6^NewYork-Presbyterian Brooklyn Methodist Hospital, Brooklyn, NY, United States; ^7^NewYork-Presbyterian Lawrence Hospital, Bronxville, NY, United States; ^8^Strategic Sourcing at New York Presbyterian Hospital, New York, NY, United States

**Keywords:** dashboard, census tracking, CRRT, HD, COVID-19, disaster planning

## Abstract

The unprecedented surge of nephrology inpatients needing kidney replacement therapy placed hospital systems under extreme stress during the COVID-19 pandemic. In this article, we describe the formation of a cross campus “New-York Presbyterian COVID-19 Kidney Replacement Therapy Task Force” with intercampus physician, nursing, and supply chain representation. We describe several strategies including the development of novel dashboards to track supply/demand of resources, urgent start peritoneal dialysis, in-house preparation of kidney replacement fluid, the use of unconventional personnel resources to ensure the safe and continued provision of kidney replacement therapy in the face of the unanticipated surge. These approaches facilitated equitable sharing of resources across a complex healthcare-system and allowed for the rapid implementation of standardized protocols at each hospital.

## Introduction

The ongoing COVID-19 pandemic placed significant stress on healthcare systems worldwide, and in particular, the massive numbers of COVID-19 patients experiencing acute kidney failure far exceeded the capacity to provide optimal kidney replacement therapy (KRT) in patients with kidney failure (1–3). Further exacerbating the surge of patients in need of KRT, there was an acute shortage of dialysis machines, both hemodialysis (HD) and continuous renal replacement therapy (CRRT), consumable supplies, personnel, and the emergence of unexpected complications such as a hypercoagulable state leading to frequent clotting and wastage of CRRT cartridges.

We previously described the strategies to deal with the surge at one of our hospitals (4, 5). We now address the tactics employed throughout our hospital network-The New York-Presbyterian (NYP) hospital system. NYP is comprised of seven hospitals and three regional hospitals with over 3,800 beds and 421 intensive care unit (ICU) beds. The number of available ICU beds more than doubled during the COVID-19 response to 901 by converting operating rooms, medical and surgical floor beds, cardiac catheterization suites, and meeting spaces to accommodate patients with ICU level needs. At the peak of the surge, 83% of our ICU beds were occupied with COVID−19 patients and with over 140 patients needing CRRT compared to the usual 30–40 across all campuses.

In response to this unprecedented surge of patients with kidney failure, we formed the **New-York Presbyterian COVID-19 Kidney Replacement Therapy Task Force** to ensure optimal care of patients requiring KRT. In this article, we report our experience with the formation and organization of this task force, and the strategies and innovative tools we developed which could inform nephrology programs faced with similar challenges (Table 1).

**Table 1:** An outline of strategies employed to optimize KRT capacity across a large institution during the COVID-19 pandemic.

**Area of intervention**	**Problem**	**Intervention**
Hospital system	• Inability to coordinate demand vs. supply of KRT equipment, disposables and personnel. Varying protocols for optimizing use of CRRT solutions • Inability to analyze date in real time	• Formation of a cross-campus task force consisting led by a nephrologist and program coordinator. Members: - Nephrologists - Nursing personnel - Supply chain representative • **Enterprise CRRT and HD Status Tracker**
Supply chain	• Major shortages of KRT equipment, and supplies	• Early (pre-emptive) action: ordered buffer fluids Ordered CRRT and HD machines • “Self” Distribution: direct delivery of supplies to hospitals or off-site • Daily monitoring of available supplies • Alternative product from non-standard suppliers
Continuous kidney replacement therapy	• Shortage of CRRT machines • Shortage of CRRT Fluid • Recurrent clotting • Shortage of personnel	• Machine sharing using 8, 12-, or 24-h shifts • Reduce fluid prescription to 15–20 mL/kg/h in stable patients • Use of **Therapy Fluid Conservation Normogram** to avoid fluid wastage • In-house preparation of CRRT Fluid using hemodialysis machines • Standardized Anticoagulation Protocol • Redeployment of perfusionists to assist with CRRT • Home dialysis nurses and technicians from external organizations assisted ICU nurses and perfusionists
Hemodialysis	• Increased load of COVID-19 patients needing isolation • Shortage of machines and dialysis schedule limitations • Shortage of nursing personnel • Prolonged exposure of nursing staff	• Use of Hepatitis-B isolation rooms and formation of COVID-19-only shifts • Reduce frequency of dialysis to twice weekly in stable patients. Transfer patients to off-site outpatient COVID-19-only HD units • Pre-emptive request to hire traveling nurses. Redeployment of nurses with prior HD experience to HD units Volunteers helped nurses and technicians with machine maintenance and patient monitoring • Use of video monitoring during HD treatment
Peritoneal dialysis	• Coordination • Catheter placement • Shortage of trained personnel	• Nephrologist champion with PD experience at each site • Surgeon willing to perform bedside catheter placement • ICU nurses dialyzed adult patients admitted to pediatric ICUs • Formation of a centralized core of PD-trained nurses to train and supervise untrained nursing staff • Perfusionists and nursing personnel without previous PD experience received PD training via PD nurses, and through external vendors (videoconferencing)

### Formation of the Inter-campus Task Force

**Figure 1:**
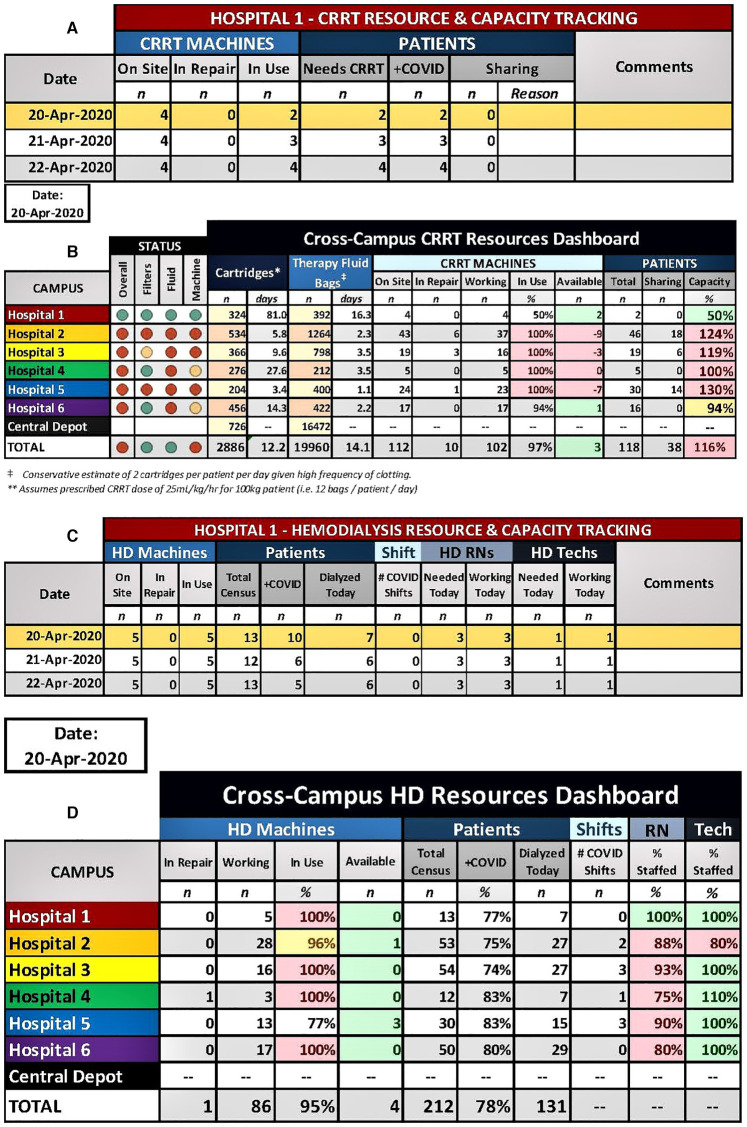
Enterprise-wide CRRT tracker **(A)** and dashboard **(B)**. Each hospital entered the details of their CRRT program **(A)**, which then automatically populated the Enterprise-wide CRRT dashboard **(B)**. The projected number of days of supply for cartridges and therapy fluid is automatically calculated based on the values of the current supply counts and based on the current patient census for each site and for the entire enterprise. Capacity calculations (CRRT patients/CRRT machines) and a color-coded status was generated for filters/cartridges, therapy fluid, and machines, which allowed for identification of hospitals that were disproportionally affected by the surge. Enterprise-wide HD tracker **(C)** and dashboard **(D)**. Each hospital entered the details of their HD program **(C)**, which then automatically populated the Enterprise-wide HD dashboard **(D)**. This allowed for quick, visual reporting of the status of working machines, census sizes, nurses, and HD technicians at each site.

In the initial phase of the surge, much attention was focused on the hospital's supply of mechanical ventilators. However, after reviewing data from other countries, hospital leadership recognized it would need to ramp up capacity for KRT exponentially in a matter of weeks. An attempt to secure additional KRT machines and supplies, described later in this report, would not provide a timely solution to the impending demand. Thus, a nephrology leader and a hospital administrator were tasked with convening a group of nephrologists and nurses from across NYP hospitals to develop a strategy to combat the impending shortage of resources. The group met daily via an online call to share local patient volume, status of CRRT machines, staffing and consumables in the face of a rapidly escalating patient census at each hospital. We used a color-based system (green, yellow, orange, and red) to indicate the status for each of these variables (Figure 1). It was immediately obvious that the hospital system was critically short on nearly all aspects of KRT support: personnel and KRT machines/disposables, and patient volume only beginning to increase. Based on daily analysis of data from a tracking tool (see section KRT Trackers and Dashboards), the group adopted a unified response plan in real time by which all KRT machines and consumables would be distributed proportionally across campuses. Existing machines and supplies were moved within 24 h between hospitals based on relative proportions of machines/disposables to patients on KRT. There would also be proportional distribution of newly acquired KRT machines. Because of this collaborative effort, all campuses were able to provide KRT proportionally during the surge period and no patient suffered from a lack of treatment.

### KRT Trackers and Dashboards

We developed a data-driven approach to analyze and guide our response to the COVID-19 surge using web-based spreadsheets. These tools are accessible to the reader (see links below).

### Enterprise CRRT and HD Status Tracker

In order to identify develop an enterprise-wide plan to support patients requiring KRT during the surge, it was necessary to know exactly the CRRT and HD censuses, machine and supply availability and staffing. To centralize this information, we developed an Enterprise-wide KRT Trackers and Dashboards for CRRT and HD. Each hospital entered the details of their own CRRT and HD programs (Figures 1A,C, respectively), which then automatically populated the Enterprise-wide KRT Dashboards (Figures 1B,D, respectively). The CRRT dashboard also included the current count of HD and CRRT disposables available at each site and at the central warehouse. The projected number of days of supply remaining for each site and for the entire enterprise based on the size of the CRRT census was automatically calculated and color-coded. The dashboard allowed for identification of hospitals that were disproportionally affected and aided in enterprise-level decisions to shift resources between hospitals or from central storage. For example, Figure 1B shows that 2 extra CRRT machines at Hospital 1 are available to be transferred to Hospitals 2 and 5 which are most in need and therapy fluid needs to be delivered to Hospital 5 from the Central Depot). Similarly, the HD dashboard allowed for quick, visual reporting of the status of working machines, census sizes, nurses, and HD technicians at each site. Both tools were critical in decision-making that occurred nightly on the cross-campus conference calls and allowed for the equitable sharing of resources across a large, complex healthcare system.

### Streamlining the Supply Chain

In anticipation of the upcoming surge of KRT patients, we had placed large orders for CRRT machines and consumables (fluids and cartridges) during February 2020. Despite early planning, we were acutely aware that we would not receive an adequate supply in time. Additionally, we were facing newly imposed allocations of consumables from our industry partners since many hospitals were vying for the same pool of supplies.

- Early (pre-emptive) Action: as part of our pandemic planning in early March we reviewed all of our key KRT supplies and successfully ordered a quantity equivalent to the previous 1 month's usage plus a 10% buffer. This buffer gave us extra time to adjust usage protocols, find alternative products on the market and work with our suppliers to determine an order release schedule. Using the same approach with KRT machines we were able to fill approximately 30% of our order in addition to rental machines.
- “Self” Distribution: Instead of using a third-party distributor to manage our dialysis supplies, we instead ordered all supplies directly to hospitals or to an off-site warehouse that was managed 24/7.This move greatly streamlined the supply process, ensuring that we had constant and open communication with our suppliers regarding estimated delivery times.
- Daily Monitoring of Available Supplies: all hospitals provided updated available stock at their respective sites every morning, which was entered in our KRT Dashboard, allowing us to accurately match inventory and estimate usage for each campus.
- Alternative Product From Non-standard Suppliers: In view of ongoing shortages, we procured significant supply of fluids from alternate vendors. However, we kept these supplies as backup since we needed to make significant practice changes prior to implementation.

### CRRT Optimization Strategies

- Use of Prolonged Intermittent RRT (PIRRT): To accommodate the rapidly increasing CRRT patient census, we instituted PIRRT instead of continuous dialysis in relatively stable patients. Consequently, two patients could be dialyzed in a shared mode using one machine alternately in 8–12-h or 24-h shifts.
- Optimization of Dialysis Prescription: We used a lower dialysis prescription of 15–20 mL/kg/h (c.f., 20–25 mL/kg/h) for those patients who could metabolically tolerate a lower dialysate dose to extend our limited fluid supply.
- In-house Preparation of CRRT Fluid: We developed and tested an in-house CRRT fluid generation strategy utilizing HD machines. The method was adapted from a previously reported protocol (6).
- Therapy Fluid Conservation Nomogram: At the peak of the surge, our CRRT fluid reserve was down to 9 days (from the usual 30 days). Partially used fluid bags at the end of the therapy were discarded, adding to the critical shortage of treatment fluids. We developed a nomogram to avoid discarded bags is described in detail in a previous publication (5).
- *Anti-coagulation Protocol:* A significant proportion of our COVID-19 patients were experiencing multiple clotting episodes while on CRRT despite therapeutic doses of systemic heparin. In collaboration with our hematologists and pharmacists, we developed an anticoagulation protocol based on anti-Factor Xa activity in patients with recurrent clotting (Supplementary Figure 1).
- Personnel: The rapid escalation of critically ill patients CRRT/PIRRT significantly added to the burden on the already depleted ICU nursing staff. We deployed perfusionists to assist with CRRT after receiving essential training. They were responsible for mobilizing equipment, and providing adjunct support to the ICU nurses. Furthermore, a large dialysis organization provided us with home HD nurses and technicians who assisted the ICU nurses and perfusionists.

### Hemodialysis

Several problems needed to be addressed for hospitalized HD patients: (1) the need for isolating COVID-19 patients, (2) the unknown but anticipated increase in patient load, (3) the risks to our non-COVID-19 population, and (4) potential staffing and supply issues (7, 8). Early in the surge, per CDC recommendations (9), we placed COVID-19-positive patients in unused Hepatitis-B isolation rooms. Subsequently, we added additional COVID-19-dedicated HD beds with partitioning shields and appropriate distancing, and eventually, we created COVID-19-only shifts. We dialyzed non-COVID-19 patients on earlier shifts, leaving the later shifts for the COVID-19 patients to allow for terminal cleaning. Twice-weekly treatments were used in stable patients at the peak of the surge. Lastly, we transferred stable COVID-19 patients to offsite outpatient COVID-dedicated HD units designated in the Emergency Management Plan. These factors made room for more COVID-19 inpatient HD treatments. All HD patients were successfully accommodated using these strategies.

In regional Hospitals without CRRT, we transferred some critically ill patients to tertiary care hospitals in the network. In the remaining patients, we performed HD on alternate days, with additional treatments for fluid and electrolyte control. Stable patients received twice-weekly HD with the occasional use of potassium binders to treat hyperkalemia.

In preparation for the anticipated surge of HD patients, we requested additional staffing including HD nurses and HD technicians during the early stages of the pandemic. We redeployed nurses with prior HD after receiving a week of refresher training on HD. Additionally, we were able to utilize volunteer research coordinators and laboratory staff for machine preparation and assisting the nursing staff. To ensure safety, all non-traditional personnel needed to be compliant with a “job profile competency task list.” To limit prolonged exposure of nursing staff we employed video monitoring in COVID-19 HD patients where treatment monitoring could be performed from outside the patient's room. These steps allowed the increase of nurse/patient ratios during the peak of the pandemic, significantly relieving the burden on our nursing staff. Additionally, because of this support, the nursing personnel reported a considerable alleviation of anxiety and stress during the surge.

### Peritoneal Dialysis

Comprehensive KRT disaster planning also included the need to establish/expand the use of peritoneal dialysis (PD) (10). Placement of PD access and the availability of PD-trained personnel were significant barriers to the widespread use of PD. We employed a multidisciplinary strategy to establish acute PD. Each site identified one key nephrologist with PD experience to coordinate urgent start PD. We identified general or transplant surgeons who could perform bedside PD catheter insertion in accordance with International Society of Peritoneal Dialysis (ISPD) guidelines (11–13). To ensure effective PD delivery, we excluded patients with extensive surgical history, ascites, active gastrointestinal issues, who required prone ventilation or had high oxygen/PEEP requirements. We treated hyperkalemic patients on PD with hybrid CRRT temporarily, or with high frequency PD prescriptions using automated PD. We also converted patients with repeated CRRT filter clotting to PD (14). A cumulative total of 22 patients received PD. At the Cornell campus, the acute PD population represented approximately 20% of the total census of KRT patients at the peak.

The redeployment of PD nurses to other areas of the hospital posed a challenge. A combination of resources to gather personnel who could deliver PD. The creation of adult ICU beds in pediatric units allowed the use of PD since pediatric nurses were trained in PD. Additionally, at one NYP site, pediatric nurses formed a rotating “Acute PD team” which allowed patients at any location to receive treatment. Additionally, training of cardiac perfusionists and bedside ICU nurses by outpatient PD nurses and from industry resources allowed expansion of the PD provider pool. The use of telehealth technology allowed these educational resources to provide “virtual hands on” training and around the clock troubleshooting support for staff at the bedside.

## Conclusion

The unprecedented surge of nephrology inpatients during the COVID-19 pandemic in New York City required us to form the New-York Presbyterian COVID-19 Kidney Replacement Therapy Task Force with intercampus physician, nursing, and supply chain representation. We organized daily meetings around novel dashboards that were developed to track supply/demand of resources and were able to make projections about the burn rate of our supplies on hand. This facilitated equitable sharing of resources across a complex healthcare-system and allowed for the rapid implementation of standardized protocols at each hospital. We have made these tools available for use given their potential benefit for nephrologists at other institutions.

## Data Availability Statement

The original contributions presented in the study are included in the article/Supplementary Materials, further inquiries can be directed to the corresponding author/s.

## Author's Note

DOI for Enterprise-wide KRT Tracker and Dashboard: https://doi.org/10.7916/d8-cvhb-4667.

## Author Contributions

All authors listed have made a substantial, direct and intellectual contribution to the work, and approved it for publication.

## Conflict of Interest

The authors declare that the research was conducted in the absence of any commercial or financial relationships that could be construed as a potential conflict of interest.
