# Supplementary material for: Optimizing Kidney Replacement Therapy During the COVID-19 Pandemic Across a Complex Healthcare System
Source: Front Med (Lausanne). 2020 Dec 22;7:604182. doi: 10.3389/fmed.2020.604182 (PMC7784642; doi:10.3389/fmed.2020.604182)

Supplementary Figure 1 - NYP Anticoagulation for CRRT Protocol

Weill Cornell Medicine | NewYork-Presbyterian | COLUMBIA

Anticoagulation Guideline During Continuous Renal Replacement Therapy (CRRT) COVID-19
Protocol for Recurrent Clotting Episodes in a 24-hour Period
Last Updated April 16, 2020

This guideline **does not apply** to the following patients

- Already on systemic heparin for another indication
- Heparin allergy
- Suspected of confirmed heparin-induced thrombocytopenia

START: **All patients should be on 500 units/hr of regional heparin.**

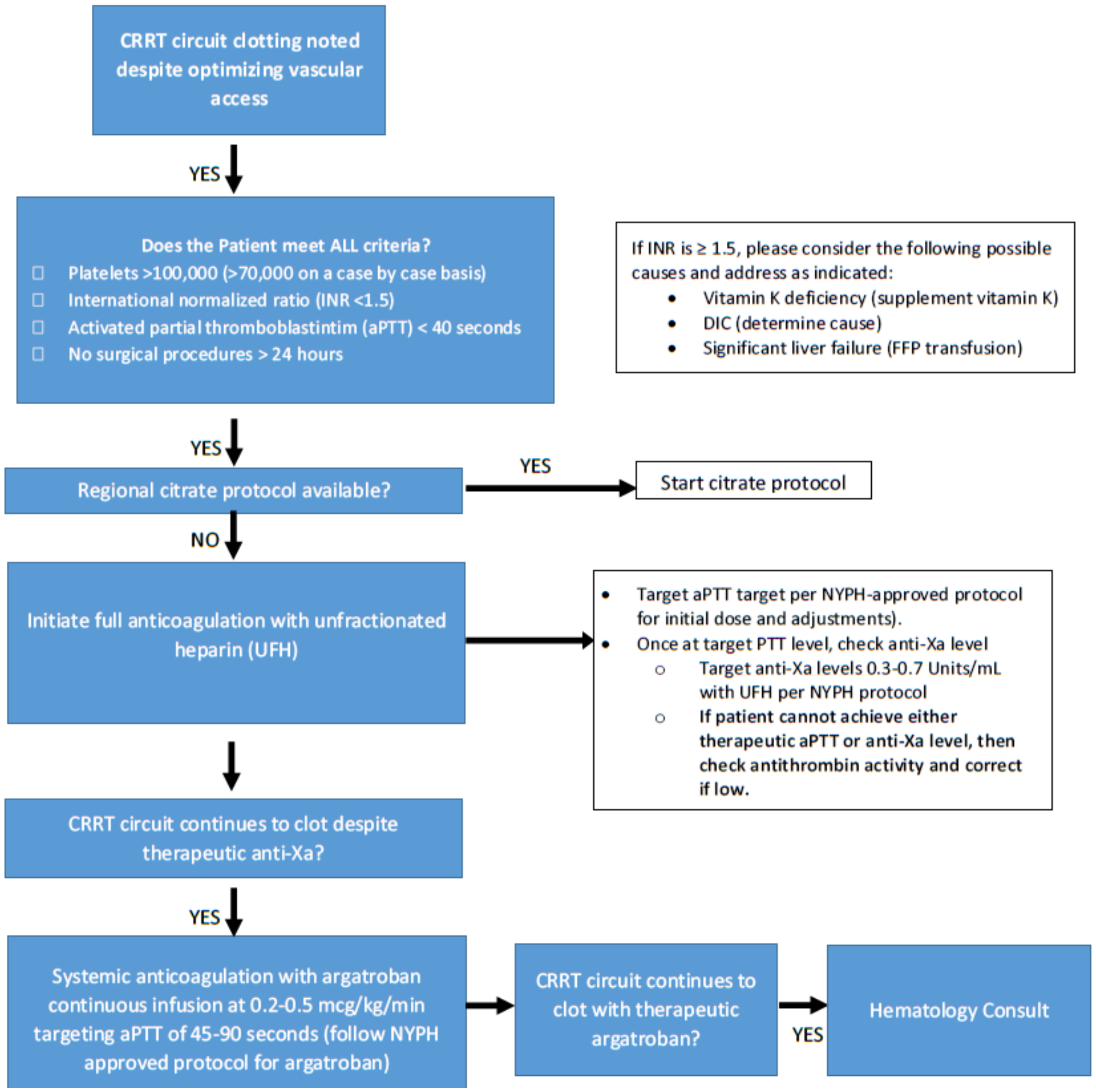

Supplement: Supplementary file 1 [file Image_1.pdf]
